# Striatal dopaminergic lesions contributed to the disease severity in progressive supranuclear palsy

**DOI:** 10.3389/fnagi.2022.998255

**Published:** 2022-08-24

**Authors:** Ming-Jia Chen, Jia-Ying Lu, Xin-Yi Li, Fang-Yang Jiao, Chuan-Tao Zuo, Jian Wang, Feng-Tao Liu, Yu-Jie Yang

**Affiliations:** ^1^Department of Neurology, National Research Center for Aging and Medicine, National Center for Neurological Disorders, and State Key Laboratory of Medical Neurobiology, Huashan Hospital, Fudan University, Shanghai, China; ^2^PET Center, National Center for Neurological Disorders, and National Clinical Research Center for Aging and Medicine, Huashan Hospital, Fudan University, Shanghai, China; ^3^Human Phenome Institute, Fudan University, Shanghai, China; ^4^Key Laboratory of Arrhythmias, Ministry of Education, Department of Medical Genetics, Shanghai East Hospital, School of Medicine, Tonji University, Shanghai, China

**Keywords:** dopamine transporter, position emission tomography, caudate, progressive supranuclear palsy, disease severity

## Abstract

**Background:**

Reduced dopamine transporter (DAT) binding in the striatum has been reported in patients with progressive supranuclear palsy (PSP). However, the relationship between striatal dopaminergic lesions and the disease severity of PSP remains to be explored.

**Objective:**

To investigate the contributions of striatal dopaminergic lesions to the disease severity of PSP.

**Methods:**

One hundred patients with clinically diagnosed PSP were consecutively enrolled in this study. The disease severity was systemically assessed using the PSP rating scale (PSPrs), and the dopaminergic lesions were assessed using the ^11^C-N-2-carbomethoxy-3-(4-fluorophenyl)-tropane positron emission tomography (^11^C-CFT PET) imaging. To explore the correlations between striatal DAT bindings and the disease severity, both the region-wise and voxel-wise analysis were adopted. Partial correlations and multiple linear regressions were performed to investigate the contribution of striatal dopaminergic lesions to the disease severity in PSP.

**Results:**

Sixty-three patients of PSP with Richardson’s syndrome (PSP-RS) and 37 patients with PSP-non-RS were finally included. The disease severity in PSP-RS was much heavier than that in the PSP-non-RS. The DAT bindings in the caudate and anterior putamen correlated significantly with the PSPrs total scores, mainly in the domains of history, mentation, bulbar, and ocular motor symptoms. The striatal DAT bindings (caudate) contributed significantly to the disease severity of PSP, independent of the motor, cognition, emotion and behavioral dysfunctions.

**Conclusion:**

Our study highlighted the independent contribution of striatal dopaminergic lesions to the disease severity in PSP.

## Introduction

Progressive supranuclear palsy (PSP) is a rare, adult-onset neurodegenerative disease, with typical clinical manifestations including impaired ocular mobility, postural instability, akinesia, and neuro-psychological impairments (Höglinger et al., 2017). The disorder severely impacts the life quality and shortens life span (Schrag et al., 1999; Nath et al., 2001). Unfortunately, there is only symptomatic treatment with mild and unsustainable efficacy available (Coughlin and Litvan, 2020). To develop effective therapies, it is of extremely high necessity to explore the factors contributing to the disease severity in PSP.

Previously, researchers have explored variable factors related to the disease severity in PSP. In cerebrospinal fluid (CSF), the levels of neurofilament light chain (Rojas et al., 2018), phosphorylated tau 181 (Rojas et al., 2018), amyloid beta 42 (Schirinzi et al., 2018) were associated with disease severity in PSP. With regard to imaging features, the bindings of several tau tracers in the midbrain were related with the disease severity of PSP (Kepe et al., 2013; Brendel et al., 2017; Perez-Soriano et al., 2017; Schirinzi et al., 2018; Malpetti et al., 2021). Additionally, other tracers to index microglial activation (Malpetti et al., 2021), synapse loss (Holland et al., 2020) and glucose metabolism (Zwergal et al., 2011), were also investigated in PSP patients.

As an atypical Parkinsonism, the dopaminergic lesions in striatum have been widely reported in PSP (Jin et al., 2013; Whitwell et al., 2017; Yoo et al., 2018; Chen et al., 2022). Compared with other tracers, the dopamine transporter (DAT) tracer represents the functional state of the dopaminergic system. The dopaminergic system plays important roles in motor control, motivation, reward, cognitive function, maternal, and reproductive behaviors (Klein et al., 2019). It’s widely accepted that dopaminergic dysfunction correlates closely with disease severity in Parkinson’s disease (Liu et al., 2018) and atypical Parkinsonism like Multiple System Atrophy (Lee et al., 2021). Given that severely decreased DAT bindings were found in both PSP-RS and PSP-non-RS subtypes (Chen et al., 2022) and the contribution of the dopaminergic lesions to the disease severity in PSP has never been explored before, we conducted this cross-sectional study aiming to explore the contribution of dopaminergic lesions to the disease severity in PSP, with the DAT positron emission tomography (PET) imaging in a relatively large PSP cohort with various subtypes.

## Materials and methods

### Participants

From January 1, 2018 to March 31, 2021, 100 patients with clinically diagnosed PSP were consecutively enrolled in this DAT PET imaging study. The diagnosis was made and reviewed by three movement disorder specialists according to the Movement Disorder Society Diagnostic Criteria (Höglinger et al., 2017). The study was approved by the Ethics Committee of Huashan Hospital and all subjects provided informed written consent before entering this study in accordance with the Declaration of Helsinki.

### Clinical assessments

The demographic characteristics of patients, including the age, sex, education, age at onset, disease duration, and levodopa equivalent daily dosage (LEDD) were systemically collected. After withdrawal of anti-parkinsonian drugs for at least 12 h, the evaluation of the PSP rating scale (PSPrs) and the motor section of the MDS unified Parkinson’s disease rating scale (MDS UPDRS-III) was made by two experienced specialists in movement disorders, reflecting the disease severity and motor dysfunctions in PSP patients, with higher scores representing more severe symptoms. Global cognition function was assessed using Mini-mental state examination (MMSE), with higher scores representing better cognitive function. Frontal behavioral abnormalities were assessed by frontal behavioral inventory (FBI), with higher scores indicating more severe abnormality. The symptoms of depression were evaluated using geriatric depression scale (GDS), with higher scores indicating greater disability.

### ^11^C-CFT PET imaging and data analysis

All participants received ^11^C-N-2-carbomethoxy-3-(4-fluorophenyl)-tropane (^11^C-CFT) PET examination on a Siemens PET/CT (Biograph 64 HD, Siemens, Germany) in a quiet and dimly lit room, within 1 month of clinical assessments. A low dose computed tomography (CT) was performed first for attenuation correction. ^11^C-CFT PET scan was performed from the 60 to 80 min after intravenous injection of 350–400 MBq of ^11^C-CFT, and reconstructed with the ordered subset expectation maximization method.

Image data were preprocessed using the Statistical Parametric Mapping (SPM) 8 software (Wellcome Department of Imaging Neuroscience, Institute of Neurology, London, United Kingdom) and subsequently processed with the ScAnVP software, version 7.0 (Center for Neuroscience, the Feinstein Institute for Medical Research, Manhasset, NY, United States) implemented in Matlab 8.4 (Mathworks Inc., Sherborn, MA, United States). Individual scans were spatially normalized into the Montreal Neurological Institute (MNI) brain space using an internal template (Bu et al., 2018). The spatially normalized PET image was then smoothed using an 8 mm full width at half maximum (FWHM) Gaussian kernel to increase signal-to-noise ratio.

Two analytical approaches were employed to explore the relationship between striatal dopaminergic lesions and disease severity. First, regional analysis was performed. The previously defined volume of interest (VOI) atlas in MNI space identifying manually with reference to the corresponding structural MR images (Bu et al., 2018) was used for semi-quantification. Tracer bindings were calculated in 7 VOIs, consisting of bilateral caudate, bilateral anterior putamen, bilateral posterior putamen (Oh et al., 2012), and occipital area. Using the occipital area as the reference region, striatal-to-occipital ratios (SORs), defined as (striatal VOI—occipital VOI)/occipital VOI were quantified for each hemisphere, and then averaged across hemispheres. Partial correlations were performed between PSPrs scores and striatal DAT binding, adjusted for sex, age at onset, and duration of the disease. The second analytical approach was voxel-wise analysis, to assess specific voxel-level correlations with PSPRrs after adjustment for sex, age at onset, and disease duration. To conduct this analysis, the preprocessed ^11^C-CFT PET images were first divided by the corresponding value in the occipital area to obtain the SOR maps. Then multiple regression model in SPM8 was used to explore the correlation. Due to hypothesis-driven confirmatory nature, an uncorrected voxel threshold at *P* < 0.01 was applied with an extent threshold of > 1-fold the expected voxels per cluster estimated in SPM.

### Statistical analysis

Potential differences between groups regarding demographic characteristics were investigated. For continuous variables, Kolmogorov–Smirnov test was performed, to investigate the normality. Independent *t*-test was performed for data of normalized distribution, and Mann–Whitney U test was performed for data of non-normalized distribution. Pearson’s chi-squared test was performed for categorical data. Partial correlations were performed, adjusted for sex, age at onset, and duration of the disease. Multiple linear regression analysis was also performed to explore the predictors of disease severity. We used SPSS (version 26.0, IBM, Armonk, NY, United States) for statistical analysis and *P* < 0.05 was considered statistically significant.

## Results

### Demographic characteristics and clinical information

In the 100 patients with PSP finally included, 63 cases (63.0%) were classified as PSP with Richardson’s syndrome (PSP-RS), and 37 cases (37.9%) as PSP-non-Richardson’s syndrome (PSP-non-RS), including 19 cases as PSP with predominant parkinsonism (PSP-P) and 18 cases as PSP with progressive gait freezing (PSP-PGF). The patients with PSP-RS took shorter disease duration (*P* < 0.01), higher disease severity as assessed by PSPrs scores (total score, *P* < 0.05; history score, *P* < 0.05; ocular motor scores, *P* < 0.05), and higher neuro-psychological symptoms by FBI scores (*P* < 0.05) and GDS scores (*P* < 0.01), than those with PSP-non-RS. No significant difference was detected between PSP-RS and PSP-non-RS in terms of age, age at onset, sex, education, LEDD, MDS UPDRS III, or MMSE. In terms of the striatal DAT bindings, similar lesions were detected between the groups of PSP-RS and PSP-non-RS. The detailed information could be found in Table 1 and Supplementary Table 1.

**TABLE 1:** Demographic characteristics and clinical information.

Variable	PSP (pooled)	PSP-RS	PSP-non-RS	*P*
	*n* = 100	*n* = 63	*n* = 37	
Sex (male/female)	62/38	36/27	26/11	0.192
Age (years)	65.92 ± 7.59	65.94 ± 7.31	65.89 ± 8.15	0.978
Age at onset (years)	62.59 ± 7.82	63.33 ± 7.64	61.32 ± 8.05	0.216
Duration (months)	34.50 (21.00, 69.75)	31.00 (19.00, 48.00)	52.00 (24.00, 86.00)	0.007
Education (years)	9.00 (6.00, 12.13)	9.00 (6.00, 14.00)	11.00 (7.00, 12.00)	0.645
LEDD	403.41 ± 327.98	400.16 ± 343.34	408.94 ± 304.57	0.898
PSPrs total score	31.00 ± 15.04 28.50 (19.25, 39.00)	33.52 ± 15.67 31.00 (22.00, 40.00)	26.70 ± 13.01 24.00 (16.00, 35.50)	0.028
PSPrs I	6.00 (3.25, 9.00)	8.00 (4.00, 9.00)	5.00 (3.00, 7.00)	0.011
PSPrs II	2.00 (0.00, 4.00)	2.00 (1.00, 3.00)	2.00 (0.00, 5.00)	0.499
PSPrs III	2.00 (1.00, 3.00)	2.00 (1.00, 4.00)	2.00 (1.00, 3.00)	0.125
PSPrs IV	6.00 (4.00, 9.00)	7.00 (5.00, 9.00)	5.00 (2.00, 8.00)	0.022
PSPrs V	4.00 (3.00, 6.00)	4.00 (3.00, 7.00)	4.00 (3.00, 6.00)	0.631
PSPrs VI	7.00 (5.00, 10,75)	7.00 (6.00, 11.00)	6.00 (5.00, 9.50)	0.134
MDS UPDRS III	41.00 (29.75, 51.00)	42.00 (30.50, 51.00)	38.00 (29.00, 52.00)	0.692
MMSE	26.00 (21.00, 28.00)	26.00 (21.00, 28.00)	27.00 (23.00, 28.00)	0.517
FBI	16.00 ± 11.16	17.89 ± 12.01	12.97 ± 8.99	0.046
GDS	10.00 (6.00, 18.00)	12.00 (8.00, 20.00)	7.00 (4.50, 13.00)	0.005
**Average DAT bindings**				
Caudate	0.73 ± 0.33	0.72 ± 0.35	0.74 ± 0.31	0.861
Anterior Putamen	0.83 ± 0.36	0.83 ± 0.37	0.82 ± 0.35	0.915
Posterior Putamen	0.49 ± 0.23	0.49 ± 0.22	0.49 ± 0.25	0.580

For continuous variables, independent t-test was performed for data of normalized distribution, given as mean ± standard deviation and Mann–Whitney U test was performed for data of non-normalized distribution, given as medium (interquartile range). Pearson’s chi-squared test was performed for categorical data. PSP-RS, PSP-Richardson’s syndrome; LEDD, levodopa equivalent daily dosage; PSPrs, PSP rating scale; UPDRS, unified Parkinson’s disease rating scale; MMSE, Mini-Mental State Examination; FBI, frontal behavioral inventory; GDS, geriatric depression scale.

### Factors correlating with progressive supranuclear palsy rating scale scores

**FIGURE 1:**
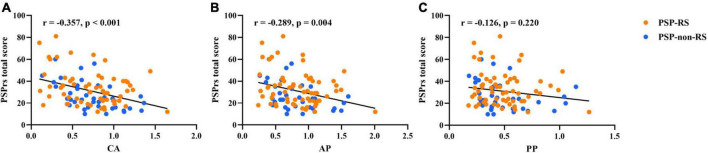
Partial correlations between regional DAT bindings [**(A)** caudate; **(B)** anterior putamen; **(C)** posterior putamen] and progressive supranuclear palsy rating scale (PSPrs) total scores in PSP (pooled), adjusted for sex, age at onset, and disease duration. Orange points, values in the PSP-RS group. Blue points, values in the PSP-non-RS group.

**FIGURE 2:**
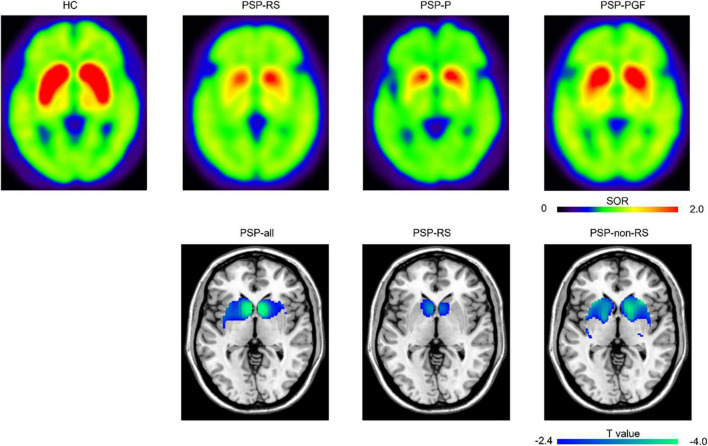
Representative ^11^C-CFT PET images and regions “survived” after the voxel-wise correlations between progressive supranuclear palsy rating scale (PSPrs) total score and DAT bindings. The top row presented the representative ^11^C-CFT PET images for healthy control and different subtypes of PSP. HC, male, 62 years old; PSP-RS, female, 61 years old, 9 months disease duration, PSPrs total score 42; PSP-P, male, 58 years old, 76 months disease duration, PSPrs total score 41; PSP-PGF, male, 77 years old, 82 months disease duration, PSPrs total score 36. The bottom row displayed the DAT bindings in specific regions showing significant negative correlations with PSPrs total score at *P* < 0.01 (uncorrected). HC, healthy control; SOR, striatal-to-occipital ratio.

In the region-wise analysis, the scores of MDS UPDRS III (*r* = 0.74, *P* < 0.001), MMSE (*r* = −0.66, *P* < 0.001), FBI (*r* = 0.66, *P* < 0.001), and GDS (*r* = 0.50, *P* < 0.001) correlated well with PSPrs total scores. The average DAT binding in the caudate (*r* = −0.36, *P* < 0.001) and anterior putamen (*r* = −0.29, *P* < 0.01), but not in the posterior putamen (*r* = −0.17, *P* = 0.22), correlated significantly with the PSPrs total scores in all patients with PSP (pooled), as shown in Figure 1. Similar correlations were also found in the subtypes of PSP-RS and PSP-non-RS (Supplementary Table 2). In the voxel-wise analysis, a significant correlation of the DAT binding in the anterior striatum (caudate and anterior putamen, *P* < 0.01) were further validated to correlate with the PSPrs total scores (Figure 2 and Supplementary Table 3).

In the further analysis with the scores of different domains in PSPrs, the DAT bindings in caudate and anterior putamen correlated well with the scores in history (I), mentation (II), bulbar function (III) and ocular mobility domains (IV) (*P* < 0.01). Meanwhile, only a weak correlation was found between the DAT binding in posterior putamen and the ocular motor dysfunctions (IV) (*r* = −0.22, *P* < 0.05). No correlation was detected between those dopaminergic lesions and the dysfunctions in limb motor (V), gait and midline (VI) (Table 2). Similar results could also be identified in the subtypes of PSP-RS and PSP-non-RS (Supplementary Table 4).

**TABLE 2:** Partial correlations between PSPrs subscores and DAT binding in PSP (pooled).

		Caudate	Anterior putamen	Posterior putamen
PSPrs I	*r*	–0.363	–0.297	–0.141
	*P*	<0.001	0.003	0.170
PSPrs II	*r*	–0.357	–0.288	–0.114
	*P*	<0.001	0.004	0.267
PSPrs III	*r*	–0.372	–0.306	–0.153
	*P*	<0.001	0.002	0.134
PSPrs IV	*r*	–0.359	–0.325	–0.220
	*P*	<0.001	0.001	0.030
PSPrs V	*R*	–0.139	–0.092	0.007
	*P*	0.173	0.369	0.942
PSPrs VI	*r*	–0.162	–0.133	–0.038
	*P*	0.113	0.195	0.712

Partial correlation coefficients were performed, adjusted for sex, age at onset, disease duration.

### Contributors to the disease severity of progressive supranuclear palsy

Given the tight association between DAT binding in the caudate and anterior putamen (*r* = 0.96, *P* < 0.001), only DAT binding in the caudate, which correlated closer with disease severity in terms of PSPrs total scores was introduced in the multiple linear regression model. It’s found that the variables of MDS UPDRS III (β = 0.395, *P* < 0.001), MMSE (β = −0.223, *P* < 0.01), FBI (β = 0.223, *P* < 0.01), DAT binding in the caudate (β = −0.217, *p* < 0.001), and GDS (β = 0.173, *P* < 0.01) contributed to the disease severity as assessed by PSPrs total scores (Table 3). In the PSP-RS group, higher MDS UPDRSIII scores (β = 0.401, *P* < 0.001), higher GDS scores (β = 0.274, *P* < 0.01), lower MMSE scores (β = −0.227, *P* < 0.05), and lower DAT binding in the caudate (β = −0.222, *P* < 0.05) were associated with higher PSPrs total scores. While in the PSP-non-RS group, higher FBI scores (β = 0.369, *P* < 0.01), higher MDS UPDRSIII scores (β = 0.333, *P* < 0.01) and lower DAT binding in the caudate (β = −0.267, *P* < 0.05) were related to higher PSPrs total scores (Supplementary Table 5).

**TABLE 3:** Multiple linear regressions predicting PSPrs total scores in PSP (pooled).

	β	*P*	*R* ^2^
Age at onset	0.039	0.516	0.747
Duration	–0.024	0.721	
Sex	0.013	0.819	
Subtype	–0.099	0.113	
MDS UPDRS III	0.395	<0.001	
MMSE	–0.223	0.004	
FBI	0.223	0.006	
GDS	0.173	0.008	
Caudate	–0.217	<0.001	

β, standardized beta-coefficient; UPDRS, unified Parkinson’s disease rating scale; MMSE, Mini-Mental State Examination; FBI, frontal behavioral inventory; GDS, geriatric depression scale.

## Discussion

As we know, this is the first study exploring the contribution of the striatal dopaminergic dysfunctions, assessed by ^11^C-CFT PET, to the disease severity of PSP. Firstly, the disease severity in patients with PSP-RS was higher than that in PSP-non-RS. Secondly, the disease severity of PSP correlated significantly with the DAT bindings in the caudate and anterior putamen, both in the PSP (pooled) and different PSP subtypes; mainly in the domains of history, mentation, bulbar, and ocular motor symptoms. Last but not least, the DAT bindings in caudate contributed to the disease severity of PSP, independent of the clinical severities of motor, cognition, emotion, and behavioral dysfunctions.

We reported that the patients with PSP-RS took a much more severe disease severity as assessed by PSPrs, especially in the domains of history (I) and ocular mobility (IV). Our findings could be supported by previous studies showing that PSPrs total scores and ocular motor scores (Choi et al., 2021) were relatively higher in PSP-RS patients than that in PSP-P and PSP-PGF, without differences in history, bulbar, limb motor, and gait/midline domains among three subtypes (Horta-Barba et al., 2021).

Severe dopaminergic lesions in the striatum were common in PSP, as shown in the DAT PET imaging (Yoo et al., 2018; Chen et al., 2022). For the first time, we reported the striatal dopaminergic dysfunctions in caudate and anterior putamen contributed to the disease severity in PSP, but not in posterior putamen. We previously reported that the lesions in posterior putamen were more prominent than caudate in PSP, reflecting specific degenerative patterns in Parkinsonism (Chen et al., 2022). Pathologically, dopaminergic projections to the striatum were from different parts of substantial nigra, with putamen from ventral part and caudate from dorsal part (Fearnley and Lees, 1991). In PSP, the ventral part of substantial nigra sustained greatest neuron degeneration (Fearnley and Lees, 1991), and the severe “floor-effect” may account for the irrelevant correlation between the DAT bindings in posterior putamen and the PSPrs total scores (Martin et al., 2008).

The contribution of dopaminergic lesions in striatum varies among different domains in the disease severity of PSP. Here, we reported the DAT binding was associated with the impairments in the domains of history (I), mentation (II), bulbar (III), and ocular motor (IV) in PSP. These domain-related correlations may be due to the involvement of dopaminergic projections in the frontostriatal-nigra circuitry regulating motor and cognitive function (Aarts et al., 2011). Previous studies supported the striatal DAT bindings related to cognitive impairments in PSP (Yoo et al., 2018). However, there was no correlation between the dopaminergic lesions and the dysfunctions in limb motor (V), gait and midline (VI). Interestingly, the items of limb motor subdomain were all eliminated in the recently modified PSPrs, due to low sensitivity to disease change (items 18, 19, 22) or failure of capturing severe disease milestones (items 20, 21, 23) (Grötsch et al., 2021). In terms of the freezing of gait and repeated falls, Cabeleira et al. (2019) reported the impairments in gait kinematics and postural control were not related to dopaminergic lesions in patients with mild and moderate PD, suggesting the involvement of some non-dopaminergic pathways. Further studies should explore the involvement of other neurotransmitters, such as acetylcholine, serotonin, norepinephrine (Maillet et al., 2012) and choline (Karachi et al., 2010; Bohnen and Albin, 2011; Rochester et al., 2012; Bohnen et al., 2013) in PSP.

So far as we know, this is the first study exploring the contributions of dopaminergic lesions to the disease severity of PSP, based on a relatively large cohort. Although our findings suggested that striatal dopaminergic lesions contributed to the disease severity in PSP, the contribution strength was only mild or moderate. This could be supported by the partial and unsustainable response to levodopa in PSP (Lopez et al., 2016). Notably, as we suggested that DAT binding contributed to disease severity in PSP, the different disease severity but similar DAT binding between the PSP-RS and PSP-non-RS groups to some extent seemed not rational. We assumed it may be not only because dopaminergic dysfunction was one of the contributing factors to disease severity in PSP, but also because its contribution was only mild or moderate in intensity. However, several limitations should be admitted here. First, our cases were enrolled by clinical diagnosis without neuropathologic verification. The misdiagnosis could not be completely ruled out. Second, the threshold we set at the SPM analysis was relatively weak that could be accepted only for the confirmative nature of the analysis. Finally, this was a cross-sectional study performed in a single center, and the center-bias should be admitted and taken with caution. Further study with expanded participants in multiple centers and longitudinal follow-up should be expected.

## Conclusion

Our study highlighted the independent contribution of striatal dopaminergic lesions to the disease severity in PSP, inspiring the further therapeutic explorations covering the dopaminergic pathways.

## Data availability statement

The raw data supporting the conclusions of this article will be made available by the authors, without undue reservation.

## Ethics statement

The studies involving human participants were reviewed and approved by the Human Studies Institutional Review Board, Huashan Hospital, Fudan University. The patients/participants provided their written informed consent to participate in this study. Written informed consent was obtained from the individual(s) for the publication of any potentially identifiable images or data included in this article.

## Author contributions

JW and C-TZ provided the data. M-JC, J-YL, and X-YL analyzed the data. M-JC, J-YL, and F-YJ wrote the manuscript. Y-JY and F-TL supervised the work and revised the manuscript. All authors contributed to the article and approved the submitted version.
