# Supplementary material for: Striatal dopaminergic lesions contributed to the disease severity in progressive supranuclear palsy
Source: Front Aging Neurosci. 2022 Aug 24;14:998255. doi: 10.3389/fnagi.2022.998255 (PMC9454812; doi:10.3389/fnagi.2022.998255)
Supplement: Supplementary file 1 [file Data_Sheet_1.docx]

Supplementary Material

## Supplementary Tables

**Supplementary Table 1** Demographic characteristics and clinical information in PSP-non-RS group

| Variable | PSP-non-RS | PSP-P | PSP-PGF | P |
| --- | --- | --- | --- | --- |
|  | n=37 | n=19 | n=18 |  |
| Sex (male/female) | 26/11 | 13/6 | 13/5 | 0.800 |
| Age (years) | 65.89±8.15 | 64.16±7.56 | 67.72±8.55 | 0.187 |
| Age at onset (years) | 61.32±8.05 | 58.74±7.33 | 64.06±8.06 | **0.043** |
| Duration (months) | 57.70±35.10 | 67.89±39.04 | 46.94±27.50 | 0.069 |
| Education (years) | 9.99±4.57 | 10.55±4.03 | 9.39±5.12 | 0.446 |
| LEDD | 408.94 ± 304.57 | 425.09±378.61 | 391.89±209.93 | 0.745 |
| PSPrs total score | 24.00(16.00, 35.50) | 26.00(21.00, 41.00) | 20.50(14.75, 27.50) | **0.029** |
| PSPrs I | 5.00(3.00, 7.00) | 6.00(3.00, 5.00) | 3.50(2.75, 5.00) | 0.086 |
| PSPrs II | 2.00(0.00, 5.00) | 3.00(0.00, 5.00) | 1.00(0.00, 2.00) | 0.118 |
| PSPrs III | 2.00(1.00, 3.00) | 2.00(1.00, 3.00) | 2.00(1.00, 3.00) | 0.893 |
| PSPrs IV | 5.00(2.00, 8.00) | 6.00(3.00, 9.00) | 3.50(1.00, 6.50) | 0.070 |
| PSPrs V | 4.00(3.00, 6.00) | 4.00(4.00, 6.00) | 4.00(2.00, 5.25) | 0.086 |
| PSPrs VI | 6.00(5.00, 9.50) | 6.00(5.00, 10.00) | 6.50(5.00, 8.00) | 0.869 |
| MDS UPDRS III | 41.16±15.46 | 42.00±18.00 | 40.28±12.73 | 0.740 |
| MMSE | 27.00(23.00, 28.00) | 25.00(20.00, 28.00) | 27.50(23.00, 28.00) | 0.233 |
| FBI | 10.00(6.00. 20.00) | 18.00(7.00, 22.00) | 9.00(5.25, 10.00) | **0.031** |
| GDS | 7.00(4.50, 13.00) | 8.00(5.00, 17.00) | 6.00(4.00, 10.00) | 0.284 |
| Average DAT bindings | |  |  |  |
| Caudate | 0.74±0.31 | 0.69±0.31 | 0.78±0.32 | 0.373 |
| Anterior Putamen | 0.82±0.35 | 0.79±0.36 | 0.86±0.34 | 0.543 |
| Posterior Putamen | 0.49±0.25 | 0.51±0.29 | 0.46±0.20 | 0.569 |

For continuous variables, independent t test was performed for data of normalized distribution, given as mean±standard deviation and Mann-Whitney U test was performed for data of non-normalized distribution, given as medium (interquartile range). Pearson's chi-squared test was performed for categorical data. LEDD, levodopa equivalent daily dosage; PSPrs, PSP rating scale; UPDRS, unified Parkinson's disease rating scale; MMSE, mini-mental state examination; FBI, frontal behavioral inventory; GDS, geriatric depression scale.

**Supplementary Table 2** Partial correlations between DAT binding and PSPrs total scores in PSP subtypes

|  |  | Caudate | Anterior Putamen | Posterior Putamen |
| --- | --- | --- | --- | --- |
| PSP-RS | r | -0.358 | -0.291 | -0.158 |
|  | P | **0.005** | **0.024** | 0.229 |
| PSP-non-RS | r | -0.442 | -0.392 | -0.131 |
|  | P | **0.009** | **0.022** | 0.460 |

Partial correlation coefficients were performed, adjusted for sex, age at onset, and disease duration.

**Supplementary Table 3** Brain regions exhibiting a significant negative correlation between PSPrs total score and DAT binding at voxel-level (P<0.01 uncorrected).

|  | Cluster level | | Peak level | | |
| --- | --- | --- | --- | --- | --- |
|  | Cluster size (mm^3^) | P value* | T value | Coordinates^#^ | Region |
|  |  |  |  | X, Y, Z |  |
| PSP (pooled) | 21880 | 0.050 | 4.36 | -8, -12, 0 | Left caudate ^a^ |
|  | 14872 | 0.080 | 4.35 | 10, 10, 2 | Right caudate ^a^ |
| PSP-RS | 11720 | 0.098 | 3.33 | -8, 12, 0 | Left caudate |
|  | 11720 | 0.098 | 3.17 | -12, 0, 18 | Left caudate |
| PSP-non-RS | 23760 | 0.034 | 3.90 | 14, 10, -2 | Right putamen ^b^ |

The extent cluster size (mm^3^) threshold of 1-fold was 9456, 8752, 2640 for pooled PSP, PSP-RS and PSP-non-RS, respectively. *, Family-wise error corrected P. #, coordinates in Montreal Neurological Institute space. a, region that showed significant negative correlation between PSPrs total score and DAT binding at voxel-level (family-wise error corrected P<0.05 and P<0.001 uncorrected). b, region that showed significant negative correlation between PSPrs total score and DAT binding at voxel-level (P<0.001 uncorrected).

**Supplementary Table 4** Partial correlations between PSPrs subscores and DAT binding in PSP subtypes

|  |  | PSP-RS | | | PSP-non-RS | | |
| --- | --- | --- | --- | --- | --- | --- | --- |
|  |  | Caudate | Anterior Putamen | Posterior  Putamen | Caudate | Anterior Putamen | Posterior  Putamen |
| PSPrs Ⅰ | r | -0.394 | -0.303 | -0.147 | -0.423 | -0.446 | -0.255 |
|  | P | **0.002** | **0.019** | 0.262 | **0.013** | **0.008** | 0.145 |
| PSPrs Ⅱ | r | -0.362 | -0.276 | -0.123 | -0.449 | -0.427 | -0.173 |
|  | P | **0.004** | **0.033** | 0.347 | **0.008** | **0.012** | 0.327 |
| PSPrs Ⅲ | r | -0.418 | -0.345 | -0.189 | -0.343 | -0.311 | -0.182 |
|  | P | **<0.001** | **0.007** | 0.147 | **0.047** | 0.074 | 0.303 |
| PSPrs Ⅳ | r | -0.269 | -0.273 | -0.247 | -0.489 | -0.395 | -0.163 |
|  | P | **0.038** | **0.035** | 0.057 | **0.003** | **0.021** | 0.357 |
| PSPrs Ⅴ | r | -0.160 | -0.130 | -0.074 | 0.005 | 0.073 | 0.248 |
|  | P | 0.221 | 0.322 | 0.576 | 0.976 | 0.684 | 0.157 |
| PSPrs Ⅵ | r | -0.239 | -0.221 | -0.117 | 0.029 | 0.031 | 0.064 |
|  | P | 0.066 | 0.090 | 0.372 | 0.873 | 0.863 | 0.721 |

Partial correlation coefficients were performed, adjusted for sex, age at onset, and disease duration.

**Supplementary Table 5** Multiple linear regressions predicting PSPrs total scores

|  | PSP-RS |  | |  | | PSP-non-RS | | |  | |
| --- | --- | --- | --- | --- | --- | --- | --- | --- | --- | --- |
|  | β | | P | | R^2^ | | β | P | | R^2^ |
| Age at onset | 0.010 | | 0.906 | | 0.723 | | 0.037 | 0.663 | | 0.789 |
| Duration | -0.086 | | 0.315 | |  | | 0.042 | 0.699 | |  |
| Sex | -0.065 | | 0.437 | |  | | 0.093 | 0.335 | |  |
| MDS UPDRS III | 0.401 | | **0.000** | |  | | 0.333 | **0.005** | |  |
| MMSE | -0.227 | | **0.041** | |  | | -0.197 | 0.076 | |  |
| FBI | 0.121 | | 0.262 | |  | | 0.369 | **0.005** | |  |
| GDS | 0.274 | | **0.003** | |  | | 0.010 | 0.913 | |  |
| Caudate | -0.222 | | **0.013** | |  | | -0.267 | **0.012** | |  |

β, standardized beta-coefficient; UPDRS, unified Parkinson's disease rating scale; MMSE, mini-mental state examination; FBI, frontal behavioral inventory; GDS, geriatric depression scale.
